# Threatened Death during Major Anesthesia, with a Brief Digression upon Shock*In the last article, page 446, eleventh line, *rima* should have been *rami*.

**Published:** 1897-10

**Authors:** Robert H. M. Dawbarn

**Affiliations:** Professor of Operative Surgery and of Surgical Anatomy, in the New York Polyclinic


### ATLANTA
### Medical and Surgical Journal.

VOL. XIV. OCTOBER, 1897. No. 8.

L. B. GRANDY, M.D., MANAGING EDITOR.

M. B. HUTCHINS, M.D., BUSINESS MANAGER.

COLLABORATORS:
A. W. CALHOUN, M.D., LL.D., VIRGIL O. HARDON, M.D., FLOYD W. McRAE, M.D., A. W. STIRLING, M.D., JOSEPH EVE ALLEN, M.D., AUGUSTA, GA., AND HENRY R. SLACK, PH.M., M.D., LAGRANGE, GA.

### ORIGINAL COMMUNICATIONS.

### THREATENED DEATH DURING MAJOR ANESTHESIA, WITH A BRIEF DIGRESSION UPON SHOCK.*

BY ROBERT H. M. DAWBARN, M.D.,

Professor of Operative Surgery and of Surgical Anatomy, in the New York Polyclinic.

II. HEART TROUBLE.

We now come to the study of the cases in which the leading or the sole factor is heart-failure; the respiration meanwhile continuing with a fair degree of regularity and effectiveness. Such instances are more often seen in chloroform- than in ether-narcosis. Sundry chloroform commissions have agreed in reporting that dogs die as a rule by respiratory failure, under chloroform. But while this is possible also in the case of human beings, surgeons know that,

*Concluded from September number. In the last article, page 446, eleventh line, *rima* should have been *rami*.

with the latter, more frequently the heart fails first. Therefore, a good anesthetist, though watching the breathing, *never removes his finger from the patient's pulse* while exhibiting chloroform. With ether this is hardly so necessary, the respiration being then the danger-signal.

*1. Inversion of the Patient.*—This, the plan of Nélaton,* should be regarded as our first choice when the heart is mainly at fault. No one could read without conviction upon this point the graphic account† of a patient upon whom, when threatened with death from chloroform, Nélaton practiced inversion three several times with final success. The first time she remained inanimate during fifteen to twenty minutes, and at length Dr. Sims believed her dead. When the pulse finally began to be noticeable and she was deemed safe, she was changed from the vertical to the horizontal posture. Instantly all signs of life ceased. Again was the patient promptly inverted, and after an apparently even more hopeless fight than before the heart resumed work. Once more the recumbent posture; again, seemingly, death; again immediate inversion. This third time Sims thought her case to be surely hopeless. But not so! And when the heart-beats were at length felt, no one dared to let her lie down until she was both wide awake and kicking vigorously.

Dr. Sims remarks that recovery after *one* inversion might not be conclusive proof; but that the *three* collapses successfully met by three inversions, in this lady's case, were to him utterly convincing as to the extreme value of Nélaton's plan—to whose coolness in this emergency he also pays high compliment.

(It is to be regretted that the lack of knowledge in those early days as to the proper technique of chloroform-exhibition, and especially as to the modes of *prevention* of such peril as that described

* The story so current, of Nélaton and the mice, is incorrect. It has this basis of fact, however, that Nélaton *Junior*, when a child, having as he supposed killed some mice with chloroform, was directed by his famous father to suspend one by the tail; whereupon it recovered. Thus Nélaton applied for his boy's instruction, a lesson from his own experience in surgery. It will be seen that this reverses the story as usually told. It is here given upon authority of the Junior Dr. Nélaton, who says that he subsequently tried this experiment upon mice "forty or fifty times or more, and always with the same unvarying result." (Dr. J. Marion Sims, "The Story of My Life," pp. 324–325.)

† Dr. J. Marion Sims, *ibid.* pp. 321-324.

above, should have induced Sims to throw the weight of his influence in America against this anesthetic.)

It would be hard to exaggerate the practical value of this plan, although the accuracy of the physiological explanation usually given by way of accounting for its effectiveness may be questioned. Thus, we commonly read that the inversion causes blood to be poured by gravity from the lowermost parts of the person into the anemic brain, which is thus stimulated to send forth more vigorous nerve-power to the failing heart. To the writer it seems plain that this is only in part the true explanation, and that, in fact, it can only apply to such blood as may be found in the neck and upper part of the chest. In only one way can blood from any point between the heart and the feet reach the brain, if the patient is inverted; *i. e.*, by being pumped through the heart. Certainly it can *not* by gravity alone. And since we know that mechanical distention of the chambers of the heart by the circulating fluid is a most important factor in exciting cardiac contraction, the proper explanation is not far to seek.

Probably no other operator has relied more fully upon this one method than has Dr. Chisolm of Baltimore; and yet although his extensive record of operations under chloroform (more than ten thousand without a death) makes his opinion deserving of respect, we think there are few who will, with him, stick to inversion alone, and refuse to use artificial respiration—in order to hasten the exit of the narcotized air—and other valuable aids to resuscitation, in addition to Nélaton's plan. Nélaton himself used artificial respiration *plus* inversion.

The writer would here urge the general use of the following method, which has several times been used successfully in his clinic, and in the employment of which his assistants are trained:

*Best Method.*—Upon the appearance of heart-failure during the anesthesia, the patient is slid down the table until his legs drop beyond its lower end, thus making a right-angle with the thighs. The table is now inverted; it is turned head downward. Consequently the patient is instantly in Nélaton's position, and his arms drop loosely on either side of his head. One assistant, only, is needed to hold the patient in place, and this is done by clasping the ankles, the legs giving an abundantly long leverage for the maintenance of the right-angle just mentioned.

Now, the operator, standing facing his inverted patient, seizes the lower part of the chest with both hands and compresses and relaxes alternately (method of von Nussbaum); his left thumb is in the intercostal space which seems most nearly over the heart (Kœnig); and the acts of compression aforesaid are very rapidly repeated—as much so as possible (Maas); thereby the more quickly pumping out poisoned air and pumping in pure air. In addition, it will be noted that following each such emptying of the lungs by the surgeon, the Nélaton posture (by dragging on the pectoral muscles, through the weight of the arms hanging by the sides of the patient's head) causes a mechanical inspiratory act after Sylvester's principle. So that at one and the same time, by the writer's simple suggestion, we can carry into effect the various treatments recommended by all five surgeons named.

This fact need not of course preclude the use of yet additional plans, if thought best, at the hands of assistants.

The method of Hill (abdominal compression, the patient lying horizontally upon the table meanwhile) which forces blood toward the heart, while obviously of some value in heart-failure is certainly less efficient for such purpose than gravity, by Nélaton's position. The same is true of tightly bandaging the lower extremities.

2. *Heat.*—It cannot be questioned that among methods of exciting a narcotized heart to resume work one of the most efficient is heat.* A quart or more of water (normal salt-solution preferred) as hot as the hand can bear, poured through a funnel, or by irrigator, into the rectum while the patient is inverted as just advised, is standard treatment. (The average capacity of the large gut, colon and rectum together, is nine pints.) Of course, if coffee is at hand, this, or other *non-alcoholic* stimulants, may be added to such enema.

The patient's body and limbs should not be left uncovered to be

*The writer places it only second in value to Nélaton's posture, and thinks it should always be used as soon as the patient is inverted.

chilled, but should be wrapped in warm blankets. This point is important and too often neglected.

Moist heat applied over the heart is of much value. The late Dr. Sands, of New York, used it repeatedly, to the writer's knowledge, who has seen not only this gentleman but several others of the best known surgeons of New York cause a blister several inches square on the precordium by application of steaming hot cloths during dangerous anesthesia-narcosis.

Dr. Charles McBurney said some years ago in a letter to the writer: "When the heart flags seriously in ether- and chloroform-narcosis I regard very hot applications directly over the heart as of decided value as a means of stimulating that organ to renewed activity. . . . I like the idea of your intravascular hot saline injections very much, and shall use it at the first opportunity."

The latter sentence has reference to a plan devised by the writer, whereby heat may with infinitely more directness and efficiency be brought to bear on the heart. This is *to introduce hot salt-water directly into the blood-current*, and thereby to stimulate both the heart and the respiratory center in the medulla.*

This is so self-evidently a useful proposition as hardly to need argument in its defense. (See article upon "Anesthesia Narcosis" in Vol. IX., *Wood's Reference Handbook*, in which the writer first suggested it.)

If, as is generally admitted, heat applied over the heart can stimulate that viscus—whether directly, by extension through the chest-wall, until felt by the apex-beat, or reflexly—how much more efficient must be that stimulation when that heat is brought directly into the heart-chambers, in the blood. The method has additional value as a heart-stimulant by increasing the bulk of circulating fluid, and thus accomplishing in a different way what Nélaton's posture does.

Four points have here to be considered: (*a*) the place of en-

*The hot enema, advised on a previous page, acts similarly, of course; being in part absorbed from the bowels by the vessels; but in an ugly case, more speedy heart-stimulation than this is called for, additionally.

trance, (*b*) the solution for injection, (*c*) the proper temperature of that solution, (*d*) the proper amount.

(*a*) In most cases, perhaps, a vein or an artery in the operation-wound will offer almost instant entrance to the canula; if not, it is the work of but a few moments to expose any superficial vein, for instance, the median basilic.

(*b*) The fluid used should be the "normal* salt solution," which is six-tenths of a part of common table-salt per hundred; roughly, a heaped teaspoonful to the quart, boiled and filtered. At the present writing this is always kept at hand during severe operations by most surgeons, for another purpose; namely, to *prevent* shock when this is feared, by using intravenous saline infusion while the patient is still on the operating-table.

[DIGRESSION UPON SHOCK.

As a brief digression, the writer begs to remark that since the autumn of 1891, this plan of *prevention* of shock has been in use at his clinic, as a consequence of some months of experimental work upon dogs, with hot saline vascular infusion after bleeding; testing intra-arterial pressures, at differing temperatures of the injected fluid, with the kymograph and the mercurial manometer, in the Columbia College physiological laboratory during the winter and spring of '90–'91. So far as known, other surgeons did not until later adopt this plan against shock.† In evidence is submitted a brief quotation from the writer's article in the *N. Y. Med. Record*, January 2d, 1892. "Whoever has seen cases of cardiac failure upon the operating-table, whether from anesthetics or from other causes, knows that surgeons rely much for resuscitation upon the application of almost (or quite) scalding hot, wet cloths over the heart. As in many such instances the patient is devoid of

* *Normal* in the sense of corresponding with the proportion of Na Cl in the blood; and not with the chemical use of the term *normal*.

Let it not be forgotten that *plain water* (devoid of Na Cl) thrown into the blood in any considerable amount is promptly fatal by disintegrating the red discs. The writer has witnessed this result, upon the dog.

† A careful study of the Index Medicus and the N. Y. Acad. of Med. library justifies this statement.

Dr. F. Lange did, however, in 1888, advocate water *by rectum* to prevent shock, where a very bloody operation was anticipated; and Drs. Weir, Tiffany and others approved.—(*Trans. Am. Surg. Ass'n.*, Vol. VI., p. 540 *et seq.*

reflexes, being completely anesthetized, I must believe that the undoubted stimulating effect of these applications is in such instances due to the direct extension of heat until felt by the cardiac ganglia.

"Whoever has noted the vigor with which unstriped muscle, everywhere, reacts to the use of heat—for example, the much stronger and decidedly more permanent uterine contractions which result from hot post-partal injections, as opposed to cold ones—must believe it probable that such unstriped muscle, forming as it does a most important tunic of the blood-vessels, would be greatly aided by *hot saline infusion* in regaining its lost tone. Perhaps, too, the central sympathetic centers would feel and respond to this stimulus.

"Now, since a more vigorous cardiac action, accompanied by a somewhat restored vascular tone, would go far toward recovery, both from hemorrhage and its attendant shock, I have felt that the experiment was well worth trying." Again, a quotation from the writer's article in the *Med. Record* of November 12, 1892: "And why would it not be well, at the end of any and every operation grave enough to make shock a probable result (though, because of the ether, not yet at hand) to inject subcutaneously a quart or two of hot salt-water? It would be painless, the patient not yet being out of anesthesia. It would certainly be harmless. And I believe it would do much to *prevent*, by maintaining filled blood-vessels, otherwise fatal shock from developing. *It seems not improbable that we shall ultimately see this done as a matter of routine, after all severe operations.*" At that date the writer had tried the method in but very few instances to *prevent* shock, using a pint or more within the vessel, and a pint or more in the cellular tissues; and of course could not speak, as all can to-day, with absolute certainty as to its value.

Where the gravity of the operation, or severe loss of blood, makes shock a probability, let the surgeon use the "ounce of prevention" by *striking first*. It he waits to treat shock until it has already struck its deadly blow at his patient's vitality, experience teaches that very likely no means at his command can then save the victim.

The writer discusses the matter here and now, both by way of further explanation as to why the hot saline infusion is of such value (against both shock and narcosis), and because it explains why the operator should always have hot salt-water at hand, for either indication.]

(*c*) The proper temperature for the injection fluid is *as hot as the hand can bear* (both for treating narcosis and for prevention of shock; upon which point see the writer's experiments, *Record*, January 2, '92, quoted). This will be about 118 degrees Fahr.* Of course the temperature at the *heart*, when the great bulk of blood has diluted the slowly entering fluid, will be much lower than this. There need be no fear of injuring the blood or other tissue by such heat. Very many times (against shock mostly, of course) the writer has now used it, as hot as the hand could tolerate, and never with cause for subsequent regret.

(*d*) The proper amount to be injected against heart-failure from anesthesia may be set down as at least one pint; a quart if the gravity of the symptoms does not abate. It must for this indication be started promptly—the patient being in Nélaton's position meanwhile—but we must avoid excessive speed of flow. Take five minutes at least, for a pint; and stop as soon as distinct improvement in the pulse can be noted. (But to *prevent shock* where there has been severe bleeding, the writer never, in the adult, uses less than a quart, intravenously, and often three pints or more, according to effect; and occasionally has to repeat this within a few hours; and in ugly cases, perhaps yet again. But almost equally efficient in prevention and treatment of shock is the long-continued hot salt-water enema *post operationem*†; the foot of the bed being elevated meanwhile.)

3. *Cold.*—Although not so efficient as heat, the application of a piece of ice over the heart, or slapping the precordium with a

*This fact has very recently been re-verified by Dr. R. C. Kemp, also in the Columbia laboratory; showing that 118-120° F. best stimulates the heart.

†This is best maintained at an even temperature, and without soiling the bed, by use of Dr. Kemp's double-current rectal tube. (Reynders & Co., N. Y.)

towel wet in ice-water, will sometimes reflexly excite more vigorous contractions. It will be useless if the patient is under anesthesia "to the surgical degree," for most of the reflexes are then abolished.

4. *Use of Drugs.*—Our fountain syringe and canula are still in place—the latter tied within a vein. Either mingled with the hot salt-water, or preceding it, or following it, we may use properly diluted *aqua ammoniæ*, a remedy of unquestionable value. The latter with strychnine and digitalis comprise all the drugs recommended as of real use in this condition by Dr. H. C. Wood, in his Berlin report in 1890; who, however, did not experiment with strophanthus and many other heart-tonics. He says: "From fifteen to twenty-five minims of the aqua ammoniæ fortior, diluted with four times its bulk of water, should be thrown directly into a vein of the arm, and repeated in fifteen minutes if necessary."

The only hypodermic abscess for which the writer has ever been personally responsible was caused by an injection of aqua ammoniæ—and not the *fortior*—in a case of extreme opium narcosis with a failing heart; not injected within a vein, as it should have been, but into the subcutaneous connective tissue of the thigh. A slough more than an inch across resulted. The same drug may be exhibited by inhalation, with distinct caution, as it is capable of causing violent local inflammation when used in excess.

Regarding *amyl nitrite*, recommended by Burrall and others, Wood found it inefficient on dogs. Paralyzing as it does unstriped muscle everywhere, and consequently completely dilating the blood-vessels, it must inevitably diminish the resistance, caused by vascular tone, against which the heart has to work in pumping blood; and is hence an indirect heart-stimulant. If that tone be lost from complete narcosis, then it is plain that in such cases, at such time, this drug would be almost useless, since its *direct* stimulant effect on the heart is but slight. Now, as Dr. Hare has pointed out, paralyzing and dilating the blood-vessels is just how chloroform kills. So that amyl nitrite may be said to do more harm than good.

*Strychnine* has already been discussed. Both as a heart and vascular tonic and as a respiratory stimulant it is of great value.

Regarding *digitalis*, the testimony is equally strong so far as the heart and vessels are concerned. The writer carries regularly to his operations a solution of strychnine sulphate in equal parts of the tinctures of digitalis and strophanthus, and has repeatedly used this combination by hypodermic needle with benefit.

*Strophanthus* alone he has not employed. However, since it is more rapid in action than is digitalis, and stimulates the heart vigorously and very much like digitalis, it would seem obviously indicated.

5. *Electricity* in the treatment of anesthesia-narcosis is mentioned rather with disapproval by most recent writers. Although the poles are applied to neck and epigastrium, to stimulate the phrenics, the current would seem as likely to stimulate the pneumogastrics and thus help to stop the heart. The writer has not personally been convinced that it does any good in these cases.

Two remaining devices the writer has never seen used; but cannot doubt that they may be of value in absolutely desperate cases. This has been repeatedly demonstrated on the lower animals. One of these is—

6. Mechanical stimulation of the heart by a long, slender needle passed through the anterior chest-wall (D. W. Buxton).*

7. The other, introduction of a finger through a cut in the precordium, thus permitting intermittent pressure on the heart through its uncut pericardium; to be repeated some twenty times, at the rate of seventy or eighty times per minute. It is not necessary to empty the heart thereby; a slight pressure will drive on some blood (G. Rowell).†

In conclusion, it is perhaps needless to say that surgeons employ not one alone but as many as are deemed wise, at once, from this list of weapons against an enemy that comes unexpectedly, and kills quickly if at all. The writer has endeavored to indicate which methods among all of these are most to be relied upon, so far as his personal experience enables him to judge.

*Anesthetics, 2d edition, 1892, p. 122.
†*Brit. Med. Jour.*, Oct. 29th, 1892.

